# Large enhancement of light extraction efficiency in AlGaN-based nanorod ultraviolet light-emitting diode structures

**DOI:** 10.1186/1556-276X-9-58

**Published:** 2014-02-04

**Authors:** Han-Youl Ryu

**Affiliations:** 1 Department of Physics, Inha University, Incheon 402-751, Korea

**Keywords:** Ultraviolet light-emitting diode, Nanorod, AlGaN, Light extraction efficiency, Finite-difference time-domain method

## Abstract

Light extraction efficiency (LEE) of AlGaN-based nanorod deep ultraviolet (UV) light-emitting diodes (LEDs) is numerically investigated using three-dimensional finite-difference time-domain simulations. LEE of deep UV LEDs is limited by strong light absorption in the p-GaN contact layer and total internal reflection. The nanorod structure is found to be quite effective in increasing LEE of deep UV LEDs especially for the transverse magnetic (TM) mode. In the nanorod LED, strong dependence of LEE on structural parameters such as the diameter of a nanorod and the p-GaN thickness is observed, which can be attributed to the formation of resonant modes inside the nanorod structure. Simulation results show that, when the structural parameters of the nanorod LED are optimized, LEE can be higher than 50% and 60% for the transverse electric (TE) and TM modes, respectively. The nanorod structure is expected to be a good candidate for the application to future high-efficiency deep UV LEDs.

**PACS:**

41.20.Jb; 42.72.Bj; 85.60.Jb

## Background

Recently, ultraviolet (UV) light-emitting diodes (LEDs) based on AlGaN materials have attracted great attention for various applications in daily lives and industry [1-4]. In particular, markets for deep UV LEDs with emission wavelengths corresponding to the UV-C (200 to 280 nm) range are expected to grow rapidly due to the increasing interests in environmental issues such as purification, disinfection, and sterilization of water and air. However, efficiency of current AlGaN-based deep UV LEDs is too low to replace UV lamps. Typically reported external quantum efficiency (EQE) of LEDs in the UV-C regions are less than 10%, which is attributed to low injection, radiative, and light extraction efficiency in deep UV LED structures. Light extraction efficiency (LEE) of AlGaN-based deep UV LEDs has been thought to be quite low owing to the strong UV light absorption in the p-GaN contact layer and the unique anisotropic optical polarization property in AlGaN quantum wells (QWs) with high Al composition [5].

Light emitted from QWs has two optical polarization modes: transverse electric (TE) and transverse magnetic (TM) modes. In the LED structures grown on a *c*-plane substrate, the polarization direction of the TE (or TM) mode corresponds to the electric field direction perpendicular (or parallel) to the *c*-axis. Therefore, the TE-polarized light propagates in both the horizontal and vertical directions. However, the TM-polarized light propagates mainly in the horizontal direction. Then, LEE of the TE mode will be much higher than that of the TM mode because the TM-polarized light undergoes strong effects of total internal reflection (TIR) due to the large incident angle on the interface of an LED chip. Consequently, LEE will decrease significantly as the contribution of the TM mode increases. In most LEDs operating in the visible and near-infrared wavelength range, TE mode emission is dominant. In AlGaN QWs, however, light is emitted as either TE or TM mode, and the portion of the TM mode increases as the Al composition increases or emission wavelength decreases [6-8]. The increasing contribution of the TM mode with decreasing wavelengths can be attributed to another cause of low LEE in AlGaN deep UV LEDs.

In order to achieve high-efficiency AlGaN-based deep UV LEDs, it is quite important to increase LEE substantially. For obtaining high LEE, several light-extracting technologies have been developed such as surface roughing [9], patterned substrates [10], and photonic crystal patterns [11-13]. However, the patterning structures have been found to be not so effective for obtaining high LEE in deep UV LEDs owing to the strong light absorption in the p-GaN layer [5]. In this research, we pay attention to nanorod structures for obtaining high LEE. Due to the nanoscale geometry, TIR inside the nanorod can be considerably reduced and light can easily escape from the nanorod structure for both the TE and TM modes. In addition, the area of the p-GaN layer can be greatly reduced, which results in the decrease of light absorption inside an LED structure and contributes to the increase in LEE [14-16].

In this work, LEE of AlGaN-based nanorod deep UV LED structures is investigated using numerical simulations. A three-dimensional (3-D) finite-difference time-domain (FDTD) method based on Yee's algorithm with a perfectly matched layer (PML) boundary condition is employed for the simulation [17]. The FDTD methods have been successfully employed for LEE simulations of vertical or nanorod LED structures [15,18,19]. Using the FDTD simulations, we calculate LEE of nanorod deep UV LED structures for both TE and TM polarization modes and investigate the dependence of LEE on structural parameters to find optimized nanorod structures for high LEE. It will be shown that LEE of nanorod UV LEDs can be greatly increased so that LEE of >50% is achievable.

## Methods

**Figure 1:**
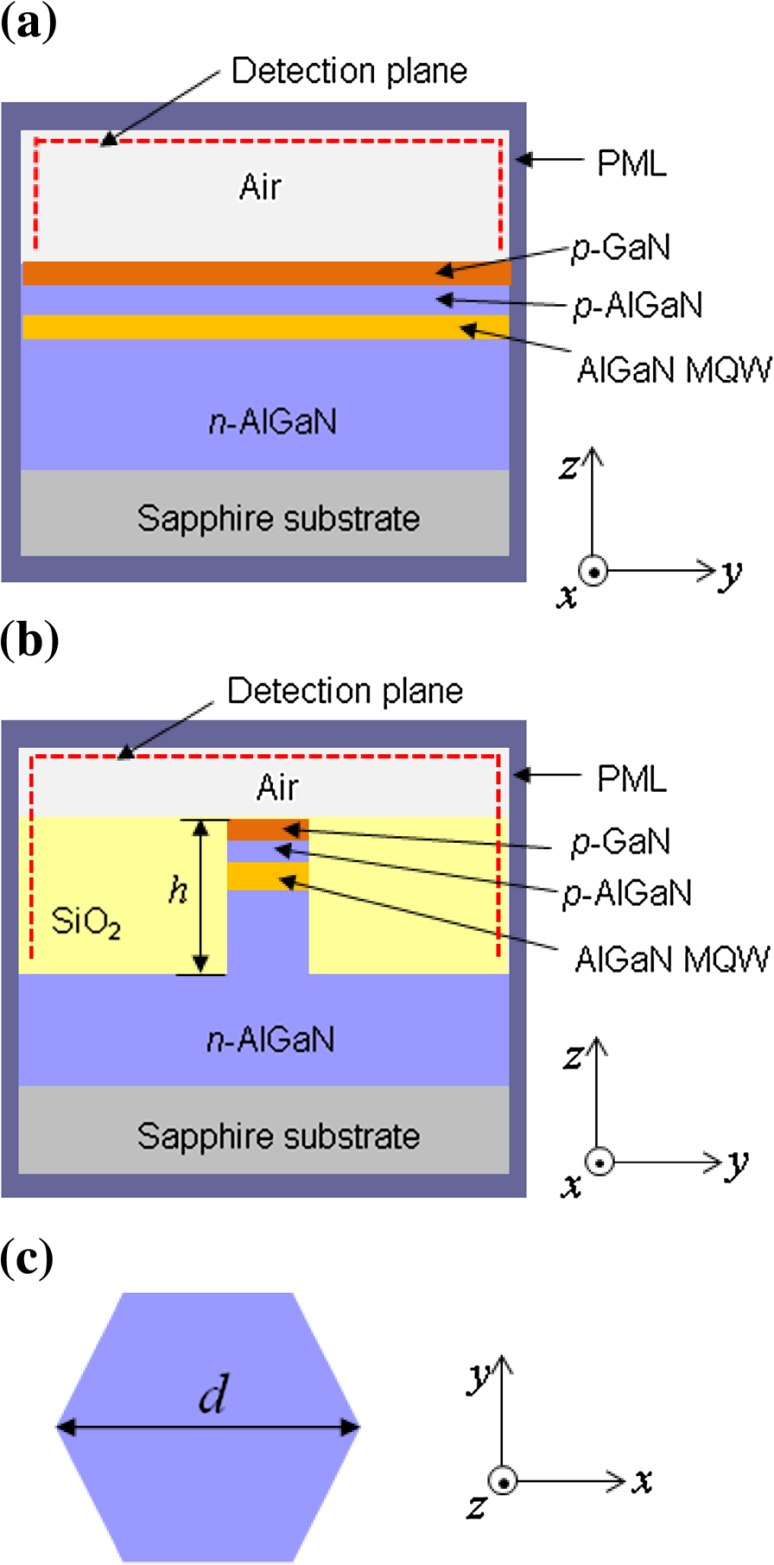
**Schematic diagram of FDTD computational domain.** Side view of the simulated LED structure is shown for **(a)** the planar LED and **(b)** nanorod LED structures. PMLs are employed for the absorption boundary condition of the FDTD simulation. The detection plane for extracted light is indicated as dotted red line. **(c)** Cross-sectional view of the simulated nanorod LED structure.

The layer structure of a simulated deep UV LED is basically similar to that of recently reported deep UV LEDs [3,4]. The layer structures are assumed to be grown on a sapphire substrate and consist of a 2-μm-thick n-Al_0.6_GaN layer, 50-nm-thick Al_0.45_GaN/Al_0.56_GaN multiple quantum well (MQW) active layers, a 50-nm-thick p-Al_0.6_GaN layer, and a p-GaN contact layer. It is assumed that the simulated UV LED chip is not encapsulated and thus exposed to air. In this work, we consider two types of LED structures: planar and nanorod structures. Figure 1 shows the cross section of the FDTD computational domain for simulated LED structures. In the nanorod LED structure, the sidewall of the nanorod is filled with SiO_2_ layers for passivation. The cross section of the nanorod is assumed to have a hexagonal shape as shown in Figure 1c because nanorod structures are mostly grown in the shape of a hexagon [16]. In the simulations, the dependence of LEE on the height (*h*) and diameter (*d*) of the nanorod structure will be investigated.

In the FDTD simulation, a single dipole source is positioned in the middle of the MQW active region. The spectrum of the dipole source has a Gaussian shape. Center wavelength and full width at half maximum of the spectrum are assumed to be 280 and 10 nm, respectively. The dipole source is polarized in the direction either parallel to the MQW plane for the excitation of the TE mode or perpendicular to the MQW plane for the excitation of the TM mode. In the computational domain shown in Figure 1, the dipole source for the TE and TM modes is set to be polarized in the *x* and *z* directions, respectively. The propagating light is completely absorbed without reflection in the PML. The Poynting vectors are calculated on the surfaces near PMLs and used to determine LEE of LED structures. LEE is defined as the fraction of emitted power out of the LED structure to the total emitted power, which is determined by the ratio of Poynting vectors integrated over extraction surfaces to total integrated Poynting vectors [18]. The plane for detecting extracted light is shown as dotted red line of the computational domain in Figure 1.

In order to obtain reliable simulation results, it is important to properly choose the refractive index and absorption coefficient of each material. The absorption coefficient of the GaN layer is chosen to be 170,000 cm^-1^[20,21]. Light is strongly absorbed in the GaN layer due to the large absorption coefficient. LEE for the planar and nanorod structures will be calculated as the thickness of the p-GaN layer varies. The absorption coefficient of the MQW layers and the n-AlGaN layer is assumed to be 1,000 and 10 cm^-1^, respectively [22]. Light extraction is also influenced by the refractive index of materials. The refractive index of GaN, AlGaN, and sapphire is set at 2.9, 2.6, and 1.8, respectively [20,22,23]. Since most of the emitted light in the nanorod structure escapes from the AlGaN layer, the refractive index of AlGaN material is expected to have a large influence on LEE results. Although the refractive index of 2.6 is used in most simulations, the dependence of LEE on the variation of the refractive index of AlGaN will be investigated in the last part of the simulation results in the next section.

## Results and discussion

**Figure 2:**
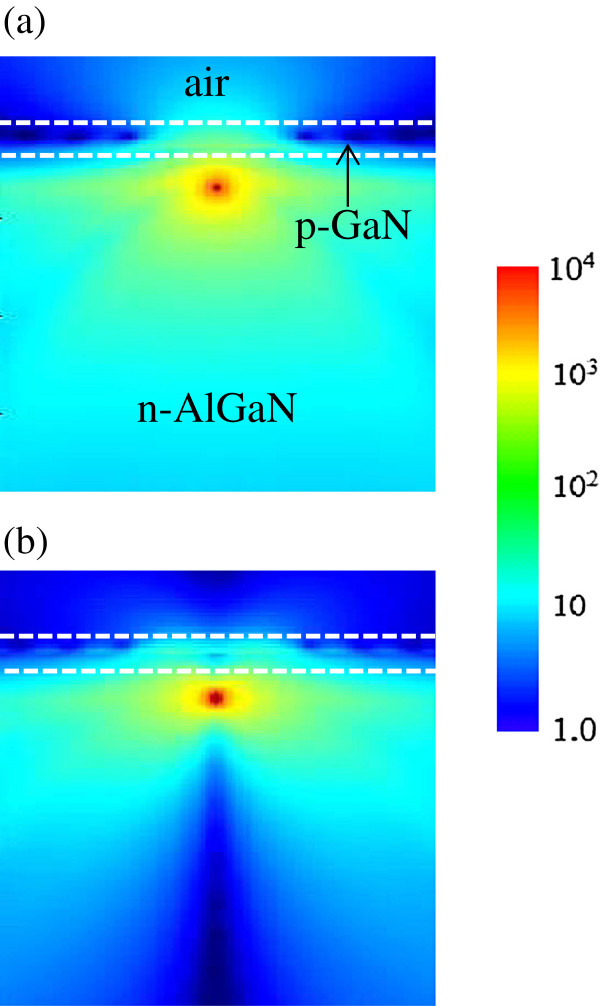
**Radiation patterns in the planar LED structure.** Electric field intensity distribution of light emitted from the dipole source is shown for **(a)** the TE and **(b)** TM modes when the p-GaN thickness is 100 nm. The color scale bar represents relative strength of electric field intensity.

First, LEE for the planar LED structure shown in Figure 1a is calculated. Figure 2 shows the electric field intensity distribution for the TE and TM modes when the thickness of p-GaN is 100 nm. The color scale bar represents relative strength of electric field intensity. In the TE mode, light can be emitted in the *y* and *z* directions because the dipole source is polarized in the *x*-axis. The light propagating in the top direction is significantly attenuated in the p-GaN layer as a result of strong UV light absorption in GaN. Therefore, only a small portion of the emitted light can escape from the LED structure, and thus LEE should be very low. For the TM mode where the dipole source is polarized in the *z*-axis, light is mostly propagating in the horizontal plane as shown in Figure 2b. In this case, it will be even harder for light to escape from the LED structure owing to the strong TIR effect in addition to the light absorption in the p-GaN layer. One can appreciate the difference of LEE between two modes by comparing the electric field intensity in air in Figure 2a,b.

**Figure 3:**
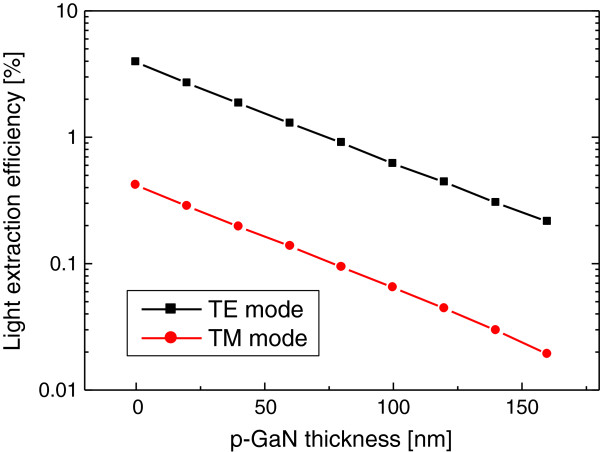
**LEE versus p-GaN thickness of the planar LED structure.** LEE is plotted as a function of the p-GaN thickness for the TE (black dots) and TM (red dots) modes.

In Figure 3, LEE is plotted as a function of the thickness of the p-GaN layer for the TE and TM modes. LEE decreases significantly as the p-GaN thickness increases. The linear dependence of LEE on the thickness in the logarithmic scale implies the exponential decrease of electric fields in the p-GaN layer. For the TE mode, LEE becomes <1% when the p-GaN is thicker than 80 nm. LEE is only approximately 4% even when the p-GaN layer is absent because of the TIR effect. LEE for the TM mode is approximately ten times lower than that for the TE mode, which is attributed to the strong TIR effect for the TM mode. Therefore, the low LEE problem of deep UV LEDs becomes even worse when the TM mode emission is dominant in the AlGaN QW. The result of Figure 3 implies that LEE of deep UV LED structures is basically limited by the TIR effect and the light absorption in the p-GaN layer.

**Figure 4:**
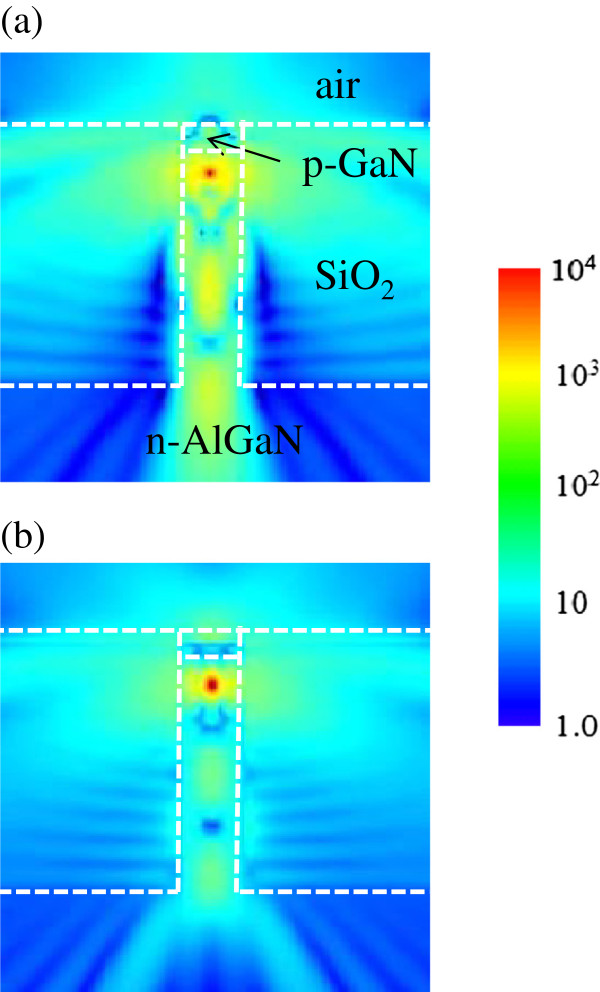
**Radiation patterns in the nanorod LED structure.** Electric field intensity distribution of light emitted from the dipole source is shown for **(a)** the TE and **(b)** TM modes when the p-GaN thickness is 100 nm. The color scale bar represents relative strength of electric field intensity.

Next, LEE for the nanorod LED structure is calculated. Figure 4 shows the electric field intensity distribution for the TE and TM modes. Here, the height and diameter of the rod are 1,000 and 200 nm, respectively. For the TE mode, light emitted in the *y* direction can be extracted from the nanorod and contribute to the large increase in LEE. However, light emitted in the *z* direction is either absorbed in the p-GaN layer or propagates in the substrate direction, which provides only a minor contribution to the LEE increase. For the TM mode, light is emitted only in the lateral directions and light propagation in the vertical direction is almost negligible as shown in Figure 4b. Therefore, the TM-polarized light can easily escape from the nanorod structure by overcoming TIR, and consequently higher LEE than the TE mode is expected.

**Figure 5:**
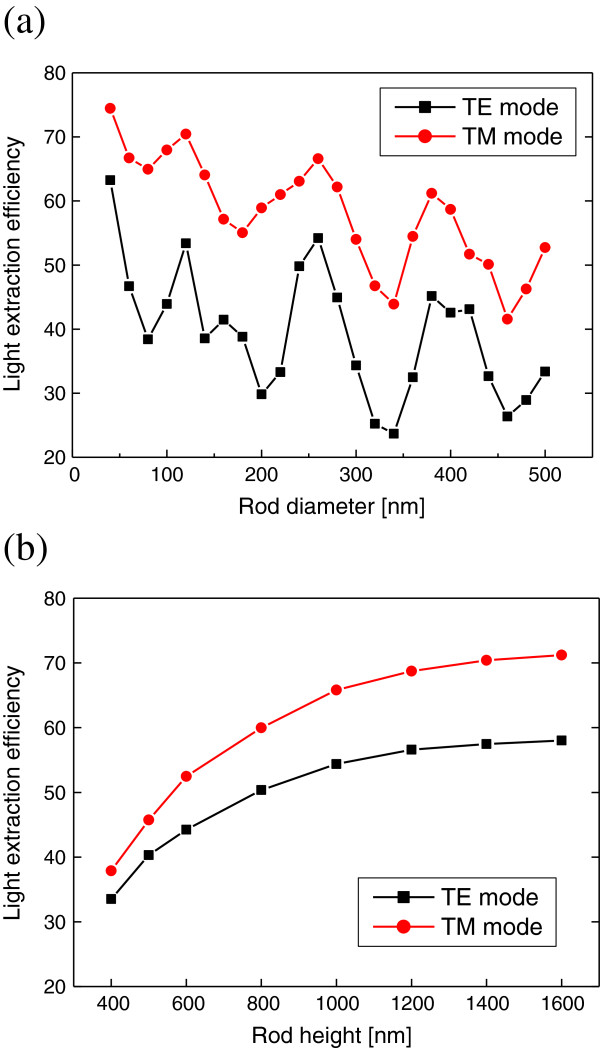
**LEE versus structural parameters of the nanorod LED structure. (a)** LEE is plotted as a function of the diameter of a nanorod when the rod height is 1,000 nm. **(b)** LEE is plotted as a function of the height of a nanorod when the rod diameter is 260 nm. Results for the TE and TM modes are represented as black and red dots, respectively.

Figure 5 shows the dependence of LEE on the diameter and height of nanorod LED structures. Here, the thickness of p-GaN layer is fixed at 100 nm. In Figure 5a, LEE is calculated as a function of the rod diameter from 40 to 500 nm when the rod height is 1,000 nm. LEE varies from 25% to 60% for the TE mode and from 40% to 70% for the TM mode as the rod diameter varies. When the nanorod LED structure replaces the unpatterned planar one, LEE is considerably increased. For the TM mode, LEE is increased from approximately 0.1% to >60%. As shown in Figure 5a, LEE for the TM mode is higher than that for the TE mode in the nanorod LED structures. Therefore, when the TM mode emission is dominant in the AlGaN QW of deep UV LEDs, the nanorod structure is expected to be a quite good solution for obtaining high LEE.

In Figure 5a, some periodic behaviors of LEE with the rod diameter are observed for both the TE and TM modes. The periodic variation of LEE is basically related with resonant modes inside the nanorod structure. When a resonant mode is formed, light is confined within the nanorod structure and cannot be easily extracted, which results in the valley of LEE in Figure 5a. Therefore, it is important to control the rod diameter appropriately to obtain high LEE. In addition to the periodic behavior, LEE globally tends to decrease with increasing rod diameter. This is because the number of confined optical modes inside the rod increases and the area of the p-GaN layer also increases as the rod diameter increases.

In Figure 5b, LEE is calculated as a function of the rod height from 400 to 1,600 nm when the rod diameter is 260 nm. In this diameter, the local maximum of LEE was obtained for both modes as shown in Figure 5a. LEE for the TM mode is higher than that for the TE mode for all values of the rod height. For both the TE and TM modes, LEE increases as the rod height increases. When the rod height is not sufficiently large, the light which escaped from the nanorod can be re-entered into the n-AlGaN layer, which results in the decrease of LEE. When the rod height is larger than 1,000 nm, LEE increases slowly and begins to saturate especially for the TM mode.

**Figure 6:**
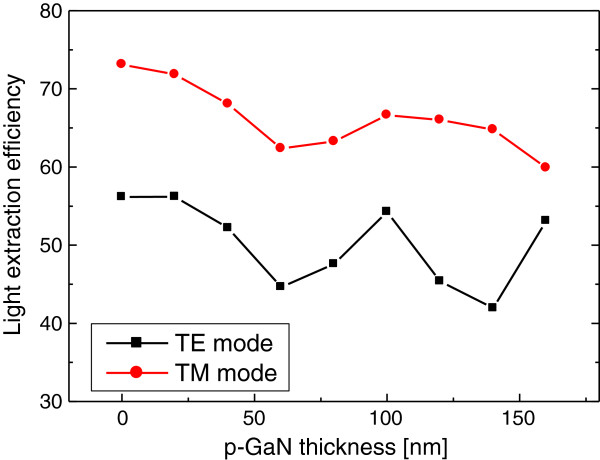
**LEE versus p-GaN thickness of the nanorod LED structure.** LEE is plotted as a function of the p-GaN thickness for the TE (black dots) and TM (red dots) modes. The diameter and height of simulated nanorods are 260 and 1,000 nm, respectively.

Next, the dependence of LEE on the thickness of the p-GaN layer is investigated to see the effect of light absorption in the p-GaN layer of the nanorod LED. Figure 6 shows LEE of the nanorod LED as a function of the p-GaN thickness. Here, the diameter and the height of nanorods are 260 and 1,000 nm, respectively. Contrary to the case of the planar LED structure in Figure 2, the decreasing behavior of LEE with increasing p-GaN thickness is not clearly observed. This is because the top-emitting light through the p-GaN layer has only a minor contribution to LEE of nanorod LED structures. However, the variation of LEE with p-GaN thicknesses is still observed. This is related with the effect of resonance modes as discussed in the results of Figure 5a. The resonant condition of a nanorod structure can be affected by the p-GaN layer thickness. The result of Figure 6 implies that the control of the thickness of the p-GaN layer is also important to obtain high LEE. In this case, the local maximum of LEE is expected when the p-GaN thickness is approximately 100 nm for both the TE and TM modes.

**Figure 7:**
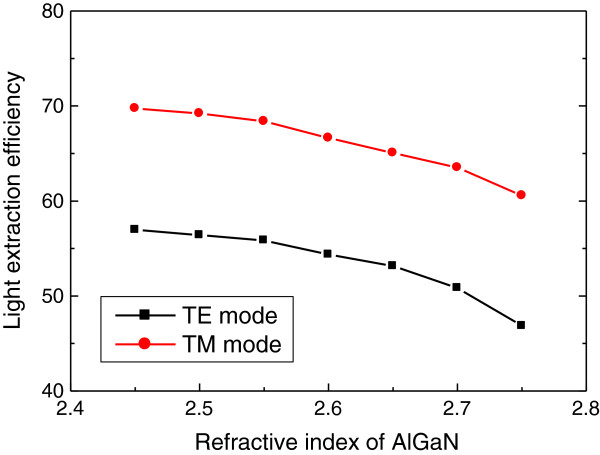
**LEE versus refractive index of AlGaN.** LEE is plotted as a function of the refractive index of AlGaN material for the TE (black dots) and TM (red dots) modes. The diameter and height of simulated nanorods are 260 and 1,000 nm, respectively.

Finally, the dependence of LEE on the refractive index of AlGaN material is investigated. Although the refractive index of 2.6 has been used up to now, there is uncertainty in the refractive index of AlGaN especially for the deep UV wavelengths. Moreover, the refractive index of III-nitride materials is generally anisotropic, which means that the refractive index can be different for each polarization. However, the optical anisotropy in AlGaN materials is not so significant; the difference in the refractive index for the TE and TM modes has been reported to be less than 0.1 in AlGaN materials [24-26]. Figure 7 shows LEE for the TE and TM modes as a function of the refractive index of AlGaN when the rod diameter and height are 260 and 1,000 nm, respectively. As the refractive index increases, LEE for both modes decreases because of the increasing contribution of the TIR effect. However, LEE decreases by only approximately 5% for both modes when the refractive index increases from 2.5 to 2.7, and LEE is still higher than 50% for the TE mode and 60% for the TM mode when the refractive index is 2.7. In addition, even when the optical anisotropy is considered, the simulation results on LEE will not change much, and LEE for the TM mode will still be higher than that for the TE mode by more than 10%.

As shown in the simulation results of Figures 5 and 6, nanorod LED structures can demonstrate high LEE that could not be obtained in other UV LED structures having the p-GaN absorbing contact layer. In particular, nanorod LED structures have great advantage for increasing LEE of the TM mode which showed very low LEE in the conventional planar LED structures. By optimizing the structural parameters of the nanorod LED such as the size of the rod and the p-GaN thickness, high LEE of >50% is expected to be achieved. Up to now, a single nanorod structure was investigated in the simulation. When the multiple nanorod structures are considered, LEE will be somewhat decreased due to the scattering and absorption in the neighboring nanorod structures. Nevertheless, still much higher LEE is expected compared with LEE of conventional UV LED structures.

## Conclusions

In this work, we investigated LEE of AlGaN-based nanorod deep UV LEDs emitting at 280 nm using 3-D FDTD simulations. Compared with the conventional planar LED structure, the nanorod LED structure showed greatly enhanced LEE even under the presence of the p-GaN absorbing contact layer. Since the TM mode emits light mostly in the lateral direction, LEE for the TM mode was higher than that for the TE mode. When the LED structure is replaced from planar to nanorod structures, LEE for the TM mode was found to increase from 0.1% to approximately 60%. In addition, LEE of nanorod LED structures was observed to have strong dependence on structural parameters such as the diameter of a nanorod and the p-GaN thickness, which could be attributed to the formation of resonant modes inside the nanorod structure. It was found that high LEE of >50% could be achieved through the optimization of the nanorod LED structures for both the TE and TM modes. The nanorod structure is expected to be a good solution for future high-efficiency deep UV LEDs especially when the TM mode emission is dominant.

## Competing interests

The author declares that he has no competing interests.
